# Primary psoas tuberculosis abscess with an iliac bone lytic lesion: a case report

**DOI:** 10.1186/s13256-022-03417-4

**Published:** 2022-05-18

**Authors:** Abdul Fattah Mohandes, Bahjat Karam, Ali Alrstom, Lugien Alasadi, Mohammad wahid Rajab Bek, Nizar Daher, Tamim Alsuliman, Raed Abouhareb

**Affiliations:** 1 Faculty of Medicine, Aleppo University, Aleppo, Syria; 2 Children’s Hospital, Faculty of Medicine, Damascus University, Damascus, Syria; 3 Infectious Disease, Internal Medicine Department, Al-Mouwasat Hospital, Damascus University, Damascus, Syria; 4 Gastroenterology and Hepatology, Internal Medicine Department, AL-Mouwasat Hospital, Damascus University, Damascus, Syria; 5 Faculty of Medicine, Syrian Private University, Damascus, Syria; 6 Service d’Hématologie, hôpital Saint-Antoine, AP-HP, Sorbonne Université, 75012 Paris, France

**Keywords:** Extrapulmonary tuberculosis, Psoas, Abscess, Ilium wing, Case report

## Abstract

**Background:**

Primary psoas tuberculosis is the presence of “Koch’s bacillus’’ within the iliopsoas muscle caused by hematogenous or lymphatic seeding from a distant site. Muscular tuberculosis has relatively low prevalence in comparison with other cases of extrapulmonary tuberculosis, which explains the difficulties in establishing the diagnosis.

**Case presentation:**

In this report, we present a challenging diagnostic case of primary psoas tuberculosis in a 38-year-old middle eastern female from southern Syria. The diagnosis was based on the clinical orientation, the observation of pulmonary lesions on the computed tomography scan, and the necrotic signs in the vicinity of the infected area. Despite the misleading primary false-negative results, the final diagnosis was reached after sufficient repetition of tuberculosis-specific testing. The patient was treated with isoniazid–rifampin–pyrazinamide–ethambutol for 2 months, then isoniazid and rifampin for 7 months, with full recovery in follow-up.

**Conclusions:**

This case highlights the importance of a clinical-based approach in the treatment of patients with psoas abscesses, especially in areas with high tuberculosis prevalence.

## Background

Psoas abscess is the presence of purulent secretions in the iliopsoas muscle compartment [1]. It has a global incidence of about 12 cases annually. In recent years, increased incidence of psoas abscesses has been noted, which can be attributed to the improvement of diagnostic imaging. Iliopsoas abscess can be either primary (without a clear source of infection) or secondary (as a result of direct spread from neighboring structures, such as vertebral bodies and the gastrointestinal tract). The vast majority are caused by *Staphylococcus aureus* infections [2, 3]. Rarely, *Mycobacterium tuberculosis* can cause a Iliopsoas abscess. The incidence of musculoskeletal tuberculosis represents 10–30% of extrapulmonary tuberculosis (EPTB) and nearly 3% of tuberculosis in general. Primary tuberculous abscess in muscles is rare, representing less than 1% of skeletal tuberculosis [4]. Psoas abscess caused by tuberculosis may be misdiagnosed due to its indolent onset and occult characteristics with nonspecific clinical presentation, which made it worth mentioning and a subject of various case reports [5, 6]. For a long period, cultures have been demonstrated to be the gold standard to diagnose EPTB. However, both normal and liquid culture suffer from some obstacles [7]. Here, we present a challenging case of primary psoas tuberculosis.

## Case presentation

A 38-year-old middle eastern married female from southern Syria (rural Damascus) was admitted to the hospital with low back pain associated with gait difficulties. The pain started 4 months earlier and radiated to the anterior sciatic region, the right groin, and the posterior region of the right thigh, unceasing during night or resting time. The pain was not exacerbated by coughing or changing posture. It was associated with tingling and redness along the right thigh. Before admission, the patient was administered intramuscular dexamethasone at a dose of 8 mg three times a week for 3 months. This was done to treat the suspected arthritis and its related back pain. The patient had no significant medical history. Physical examination revealed tenderness in the right paraspinal muscles, associated with right hip movement limitation, while the proximal right thigh strength could not be evaluated due to excessive pain, and no swelling was found above the inguinal ligament. The rest of the examination was within normal limits. Laboratory tests were normal apart from white blood cell count of 12,000/µl, neutrophils 89%, hemoglobin 10 g/dl, mean corpuscular hemoglobin (MCV) 70 fl, erythrocyte sedimentation rate (ESR) 87 mm/h, and C-reactive protein (CRP) 86.7 mg/l (reference range (RR) up to 6 mg/l). Human immunodeficiency virus (HIV) test was negative, urinalysis was normal, and the Quantiferon-TB test was within normal range, being considered as an exclusion test in immunocompetent patients in an endemic country.

**Fig. 1:**
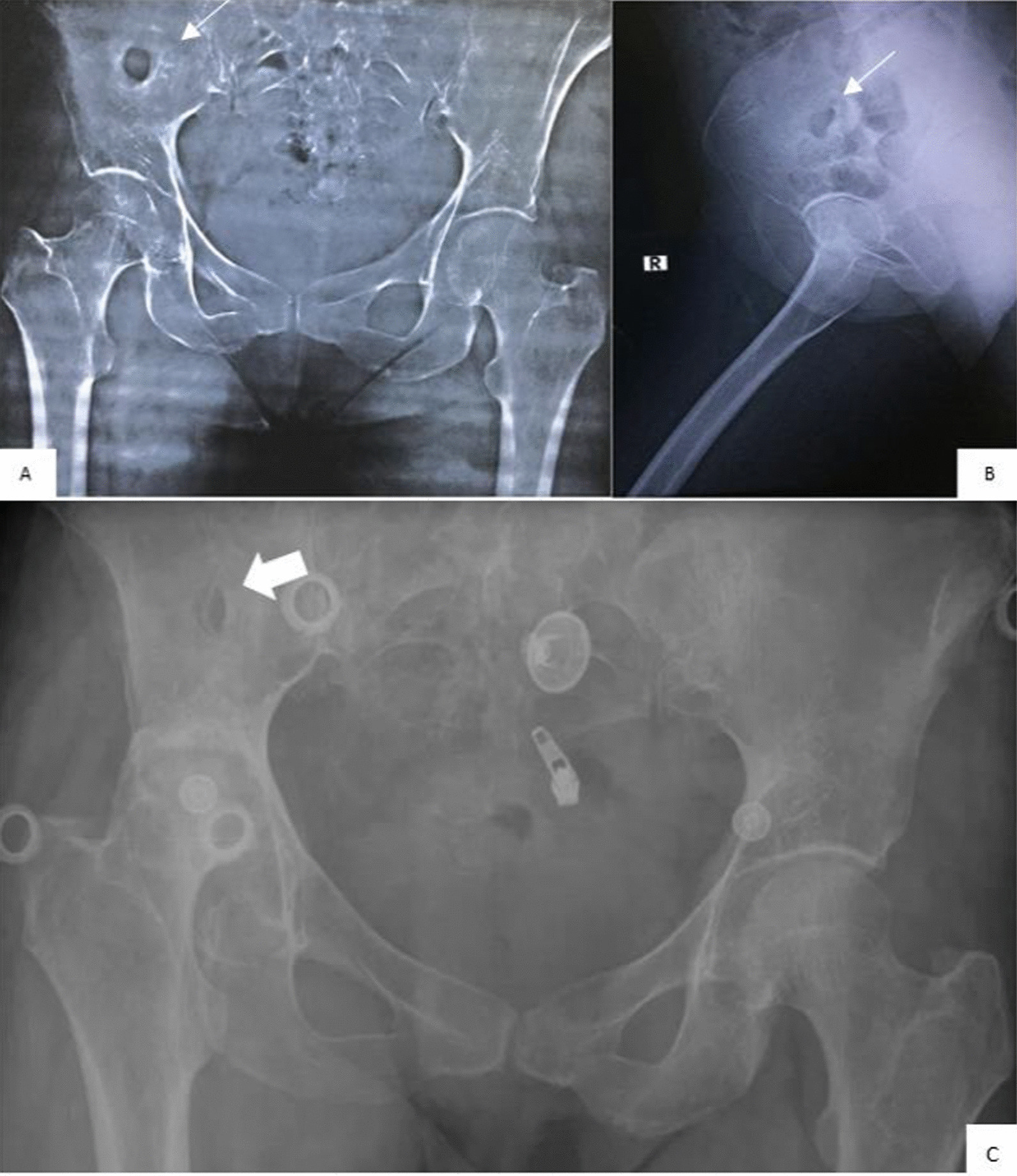
Posteroanterior (**A**) and side view (**B**) of the pelvic, showing necrosis in the right iliac wing (white arrow). **C** Posttreatment follow-up X-ray showing the remaining necrosis in the right ileum wing (white arrow)

**Fig. 2:**
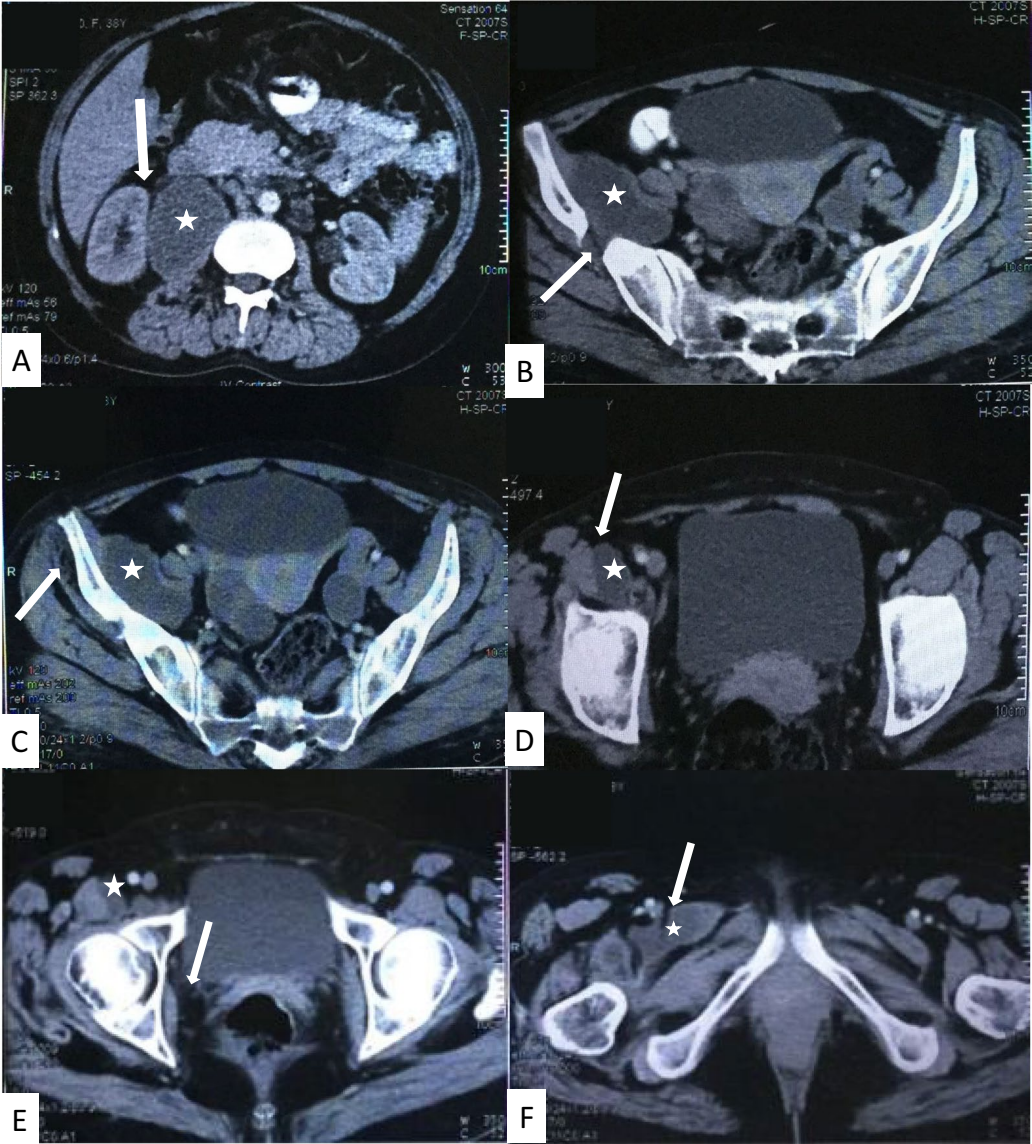
Abdominal and pelvic CT scan showing a transverse section in the Iliopsoas abscess (white star) with (**A**) the right kidney slightly pushed anteriorly (white arrow), (**B**) necrosis of the right ileum wing (white arrow), and (**C**) abscess in the right gluteal muscles (white arrow). The abscess extended to the right piriformis muscle (white arrow) (**D**) down to the level of the right obturator muscle (white arrow) (**E**), and the right femur adductor muscle (white arrow)(**F**)

Although there was no history of previous trauma, and also considering that the iliac bone is an unlikely site of hematogenous seeding, the pelvic X-ray showed remarkable necrosis in the wing of the right ilium (Fig. 1). Abdominal computerized tomography (CT) showed that the right kidney was slightly pushed anteriorly due to an abscess in the right psoas muscle measuring 7 × 4 cm^2^ and extending to the right femur adductor muscles, the right obturator muscle, and the right piriformis muscle, with another abscess in the right gluteal muscles as shown in Fig. 2.

**Fig. 3:**
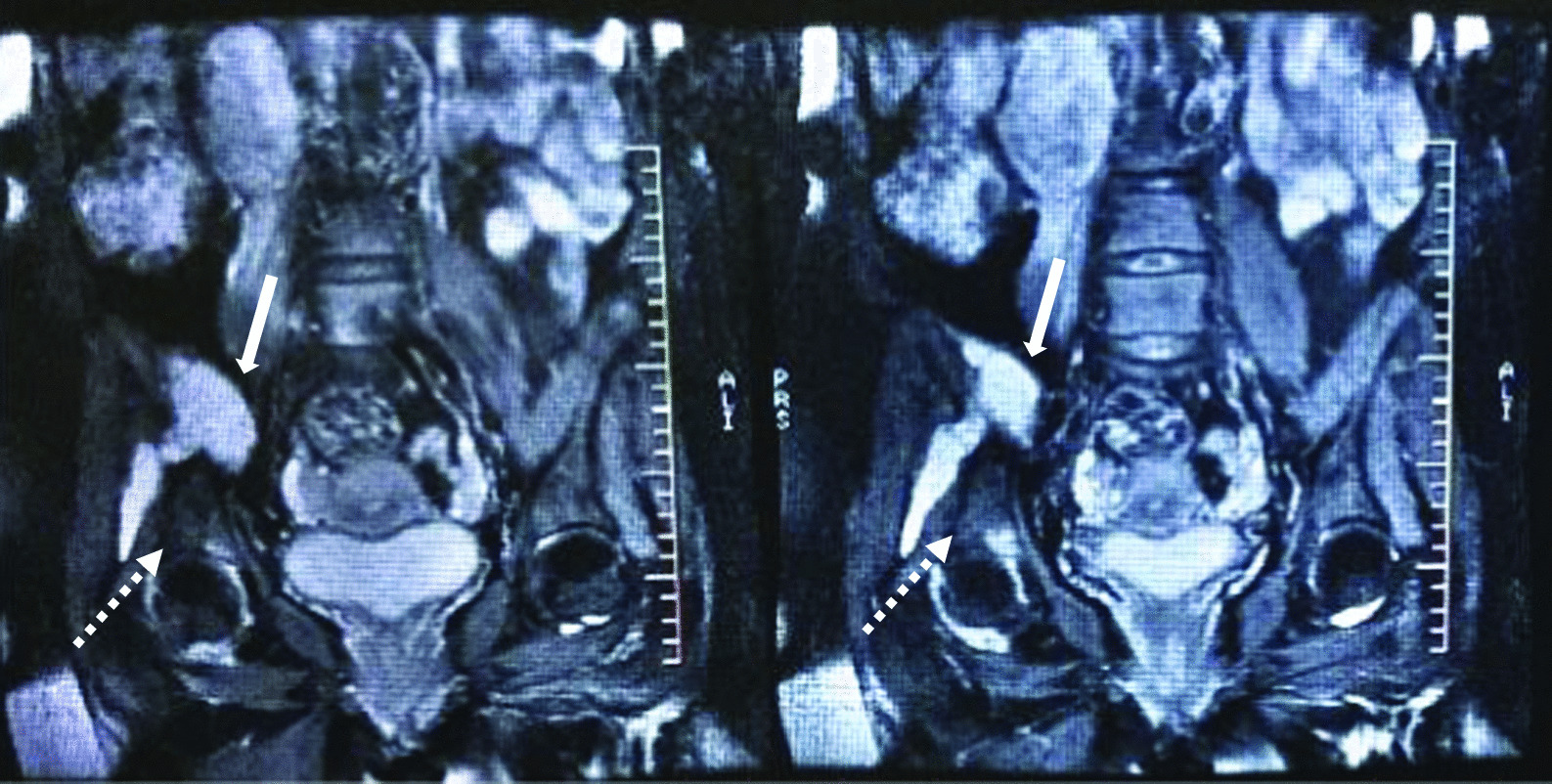
T2 abdominal and pelvic MRI showing a cyst above the right iliac bone (white arrows) extending through it, necrosis of the ilium wing, and joint subluxation between the right head of the femur and the acetabulum due to osteomyelitis (dashed arrows)

Magnetic resonance imaging (MRI) demonstrated a cyst above the right iliac bone, necrosis of the wing of ilium, and joint subluxation between the right head of the femur and the acetabulum suggesting osteomyelitis (Fig. 3).

**Fig. 4:**
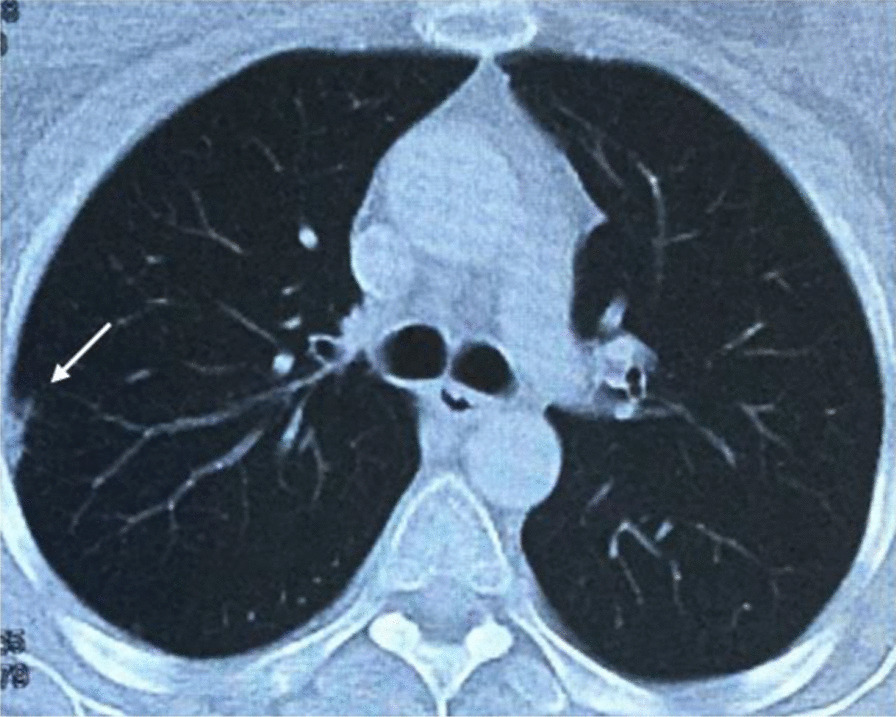
Chest CT scan showing a peripheral nodule in the lower lobe of the right lung (white arrow)

Contrast chest CT scan was done to extend the pathology workup and determine whether the abscess was primary or secondary. The scan showed a peripheral nodule in the lateral upper lobe of the right lung with collapse of the lateral segment in the inferior lobe of the right lung (Fig. 4). Both CT scan and X-rays revealed no evidence of vertebral destruction.

A CT-guided biopsy of the abscess showed bloody fluid that contained leukocytes and phagocytes with accumulations of scantily epithelioid histiocytic clusters suggestive of granulomatous tuberculosis. No neoplastic changes were seen. Gram staining on the same sample was negative, and bacterial culture was sterile. Ziehl–Neelsen stain and TB culture on Löwenstein–Jensen medium for 60 days were also negative twice.

A new sample from the iliopsoas lesion was obtained by surgical ablation. The histopathological examination concluded the diagnosis of granulomatous tuberculosis, and Xpert-MTB test came back positive on the second repetition. As a result, the patient was treated with (isoniazid 300 mg daily, rifampin 600 mg daily, pyrazinamide 1600 mg daily, and ethambutol 1100 mg daily) for 2 months, followed by isoniazid and rifampin for 7 months. The patient recovered by the end of the planned treatment with normalization of ESR and CRP values. Necrotizing of the right ileum was the only remaining damage (Fig. 1C).

## Discussion

Our case represents a primary TB abscess as we could not detect any discitis or vertebral destruction in preformed imaging. Primary psoas abscess is more common in children and young adults. It could occur as a result of hematogenous or lymphatic spread from a far separate site. Risk factors include intravenous drug use, diabetes, renal failure, human immunodeficiency virus (HIV) infection, and other forms of immunosuppression [8]. In Asia and Africa, 99% of iliopsoas abscesses are primary, while only 17–61% are primary in Europe and North America. More than 88% of primary iliopsoas abscesses are caused by *Staphylococcus aureus*. *Mycobacterium tuberculosis* is, however, an uncommon cause in developed countries compared with developing countries [1]. In one Indian study, 2 (5.1%) out of 39 children with bone TB had psoas abscess [9], while in a case series from Germany that included six patients with psoas abscess secondary to spinal infection, only one patient had TB as the cause of psoas abscess [8]. Reports about primary TB psoas abscess being the presenting manifestation without evidence of active TB infection elsewhere at the time of diagnosis, as in our case, are even less frequent [10]. Turkey is a neighboring country to Syria and is estimated to have a similar TB incidence (10–49 per 100,000) [11]. A recent report from this country included 949 patients with tuberculosis who were followed for up to 16 years. Of those, only two patients had iliopsoas TB infection, with no information regarding whether it was a primary or secondary iliopsoas TB infection [12].

To the best of our knowledge, this is the first primary TB psoas abscess to be reported from Syria.

Generally, diagnosing primary tuberculous psoas abscess is challenging [5]. In this case, the patient presented without fever, which may have been suppressed due to corticosteroid treatment, and which made the diagnosis harder. Nevertheless, the presence of a pulmonary lesion, case location in a developing country, and the suspected immunosuppression are the reasons making tuberculous psoas abscess highly suspected. Bacterial culture with Gram and Ziehl–Neelsen stain help to demonstrate the etiological organism. While CT scan is nowadays the optimal radiographic modality to evaluate a psoas abscess, MRI can provide improved definition of soft tissues and adjacent structures. In our patient, pelvic CT scan and MRI demonstrated the presence of a right psoas abscess with wide extension to the right femur adductor muscle and other surrounding structures. However, the CT-guided biopsy of the abscess was negative, and the bacterial culture was sterile. Ziehl–Neelsen stain, TB culture, and Xpert-MTB were also negative. Necrotizing of the ilium’s wing, the involvement of adjacent structures, the absence of response to treatment with antibiotics, and the unidentified pathogen were all indications for surgical drainage. Because of the high clinical suspicion, we repeated the diagnostic tests twice (Xpert-MTB—Ziehl–Neelsen staining-Gram stain-bacterial culture) and obtained a positive Xpert-MTB test for tuberculosis only on the second repetition, which emphasizes that false-negative results in suspected cases must be considered. This is explained by the highly volatile sensitivity of the Xpert-MTB/RIF test that ranges from 25% to 96.6% [7]. Several papers have reported psoas abscess due to TB infection [5, 13]. However, this is, to the best of our knowledge, the first to cause necrosis in the ilium wing. Moreover, this case emphasizes the importance of TB screening in patients from endemic war-torn areas, where healthcare services may be of mediocre quality, before starting long-term corticosteroid treatment or any other immunosuppressive medication. Our patient lived in rural Damascus, an area that had an increased number of TB cases with limited access to healthcare facilities during the conflict years [14]. Therefore, it is of great importance to mention that the long-term treatment with dexamethasone for misdiagnosed arthritis may have resulted in further insidious extension of the abscess in this patient.

## Conclusions

High clinical suspicion and thorough analysis of the patient’s medical history play a vital role in diagnosing extrapulmonary tuberculosis as a rare cause of lower back pain, especially in prevalent areas, despite false-negative test results and a misleading presentation.

## Data Availability

All the data of this case report are available on request.
